# Impacts of Oral Florfenicol Medication and Residues on the Kidney and Liver of Nile Tilapia *Oreochromis niloticus* (L.)

**DOI:** 10.3390/vetsci10010036

**Published:** 2023-01-03

**Authors:** Avishek Bardhan, Thangapalam Jawahar Abraham, Jasmine Singha, Ravindran Rajisha, Edaparambil Krishnappan Nanitha Krishna, Satyen Kumar Panda, Prasanna Kumar Patil

**Affiliations:** 1 Department of Aquatic Animal Health, Faculty of Fishery Sciences, West Bengal University of Animal and Fishery Sciences, Kolkata 700094, India; 2 Fish Processing Division, ICAR—Central Institute of Fisheries Technology, Willington Island, Cochin 682029, India; 3 Aquatic Animal Health and Environment Division, ICAR—Central Institute of Brackishwater Aquaculture, Chennai 600028, India

**Keywords:** florfenicol-feeding, serum biochemistry, antibiotic residues, hepatotoxicity, renal toxicity, histological aberrations

## Abstract

**Simple Summary:**

Florfenicol, an approved aquacultural antibiotic, is extensively used in temperate countries as therapeutics against several fish bacterial pathogens. It has never been introduced or used in several tropical countries. In earlier safety studies in tropical conditions, several alterations in the biological responses of Nile tilapia were documented. Yet, its safety was proclaimed at the juvenile stage. Though histopathological changes in the kidney and liver tissues upon oral florfenicol medication were assessed, the residue accumulation and depletion in these tissues were not documented vividly. The current study was undertaken to look at the biological responses, residue deposition and developmental oxidative stress in Nile tilapia upon oral administration at 0–10 times the therapeutic dose (15 mg kg biomass^−1^ day^−1^) for 10 consecutive days and observed for 43 days post dosing. The therapeutic dose group documented 100% survival. Alterations in serum biochemistry were noted, which recovered completely with the suspension of medication. Florfenicol residues peaked on day 10 of medication and were undetectable within 9 days of post-dosing. Oral medication even at the therapeutic dose caused reversible oxidative stress. The results provided an insight into how the florfenicol residues behave in the vital organs at higher temperatures under controlled use in tropical conditions.

**Abstract:**

Florfenicol (FFC), an approved aquaculture antibiotic, is administered in feed at doses of 10–15 mg kg biomass^−1^ day^−1^ for 10 successive days. In this study, healthy *Oreochromis niloticus* were fed with 0–10 times the therapeutic dose of 15 mg kg biomass^−1^ day^−1^ for 10 days and tracked for 43 days post dosing. Assessments of residue accrual and depletion, oxidative stress, serum biochemistry, histopathology and extent of kidney and liver damages were made. FFC dosing reduced the feed intake significantly. The therapeutic dose produced no mortalities on day 10. Dose-dependent alterations in serum biochemistry were noted upon dosing. Several histopathological alterations were observed in the kidney and liver, which vindicated the toxic potentials of FFC. The residual FFC and florfenicol amine (FFA) accrual, depletion and oxidative stress responses, such as increased malondialdehyde, total nitric oxide, ferric reducing antioxidant power and reduced glutathione S-transferase activity, were documented. The dietary FFC persuaded the physiological state of *O. niloticus*, the effects of which normalized sparsely with time upon cessation of dosing at the higher doses. The study provided a brief outlook on the physiological responses upon oral FFC administration, which should be kept in mind during its application for fish health safety purposes.

## 1. Introduction

Florfenicol (FFC), a synthetic analogue of thiamphenicol, is one of the most recent additions to the USFDA-approved aquaculture antibiotic arsenal for incorporation into fish feed as a type A medicated article [1]. Florfenicol has significant activity against a broad spectrum of Gram-negative bacteria because of its molecular structure [1]. The benefit of increased productivity from the use of veterinary medical products (VMPs) including antibiotics in aquaculture does not come without the risk of drug residues remaining in the treated farmed fish tissues or residues in fish products, which constitute a health risk to consumers [2,3]. Furthermore, the widespread and unregulated use of antibiotics in aquaculture has resulted in various issues related to antibiotic resistance [2]. Antibiotics are toxic to fish if abused. The toxicological effects of antibiotics on different organs of the host fish are still not fully understood or evaluated [4]. In intensive tilapia cultivation, FFC has often been recommended as a therapeutic agent to control several bacterial diseases [5]. It is one of the most commonly used antibiotics in major aquaculture-producing countries, such as Chile, China, Vietnam, South Korea, Norway, Egypt and Brazil [2]. Although reports are available on the use of FFC to control bacterial diseases in finfish aquaculture elsewhere [6,7,8,9], the effects and safety of oral administration of FFC in tropical fish have not been well studied. Our recent safety studies demonstrated that FFC is well tolerated, suitable and safe for Nile tilapia *Oreochromis niloticus* juveniles, with reversible physiological and biochemical alterations at the therapeutic dose and dosage of 10–15 mg kg biomass^−1^ day^−1^ for 10 consecutive days [10,11,12].

FFC-induced toxicity has rarely been assessed and recorded in various fish organs of tropical fish. According to Limbu et al. [2], FFC is less toxic because of the absence of a nitro group, and it is used extensively in veterinary medicine, in contrast to chloramphenicol and thiamphenicol. However, their side effects cannot be ignored, as residues of these drugs may pose a health risk to consumers [2] and the host fish [10,11]. Thus, it is of great importance to guide their proper use to guarantee food safety and animal welfare. The current study was, therefore, undertaken to illuminate and explore the extent of kidney and liver toxicity in healthy *O. niloticus* juveniles upon dietary FFC administration extended up to 10 times the maximum therapeutic dose under tropical Indian conditions, while keeping the dosage period constant, i.e., 10 consecutive days. The study also interrelated the physiological responses of organ toxicity with oxidative stress, serum biochemistry and histopathology upon FFC administration.

## 2. Materials and Methods

### 2.1. Ethics Statement

The experimental procedures were performed under the All-India Network Project on Fish Health, following the guidelines of the Indian Council of Agricultural Research, New Delhi (CIBA/AINP-FH/2015-16). The guidelines of the Committee for the Purpose of Control and Supervision of Experiments on Animals (CPCSEA) for Experimentation on Fishes [13] were all adhered to.

### 2.2. Experimental Fish and Acclimation

Healthy *Oreochromis niloticus* juveniles (13.31 ± 0.23 g and 9.10 ± 0.63 cm) were obtained from a grow-out farm (Lat 22°27′50.2158″ N; Long 88°23′7.4004″ E) in West Bengal, India. Acclimatization for 15 days at 25 ± 2 °C in 500 L-capacity, fibreglass, reinforced plastic (FRP) tanks at 60 juveniles/tank was performed. The floating pelleted feed of 2 mm diameter (Aquaxcel, Cargill, Maharashtra, India), comprising 8% fat, 42% protein, 5% fibre and 11% moisture, was offered to the juveniles at 2% body weight (BW) 3 times a day.

### 2.3. Experimental Setup and Dosing Protocol

A total of 15 FRP tanks were arranged, each containing randomly collected 50 juveniles from the acclimatized stock. These asymptomatic and healthy juveniles were further reacclimatized in the respective tanks for 7 days (pre-dosing tenure). The juveniles were allotted into 5 groups, viz., group 1: 0× control, group 2: 1× (15 mg kg biomass^−1^ day^−1^), group 3: 3× (45 mg), group 4: 5× (75 mg) and group 5: 10× (150 mg) in triplicates. Once in 3 days, about 50% of the water was exchanged to remove wastes and faeces. The basic water quality criteria, such as temperature (23.00–29.00 °C), pH (7.70–8.40), dissolved oxygen (4.80–5.32 mg L^−1^), ammonia (0.002–0.007 mg L^−1^), nitrite (0.13–0.52 mg L^−1^) and nitrate (0.13–0.54 mg L^−1^), were examined twice a week and maintained optimally.

### 2.4. Florfenicol Diet Preparation and Dosing Administration

To produce an estimated dosage of 0–150 mg kg biomass^−1^ day^−1^, the inclusion rate for florfenicol (Tokyo Chemical Industry, CAS RN: 73231-34-2; Product Number: F0811-5 g, Tokyo, Japan) was computed. Vegetable oil (5 mL kg feed^−1^) and the desired amount of FFC were combined, and the resulting emulsion was then utilized for top-coating in increasing sequence of concentration. The 60-day study had three phases: acclimation for 7 days, FFC-dosing (FD) for 10 days and post-FFC-dosing (PFD) for 43 days. Throughout the pre- and post-dosing periods, the juveniles were fed with control feed. The corresponding FD groups received the designated FFC diets for 10 days. For a given day, a feed ration at 2% BW was distributed during the morning, noon and evening equally. After each feeding, any food that remained in the tank 60 min later was siphoned out, gathered tank-by-tank each day, dried overnight and weighed. The vivid descriptions of the preparation of medicated and control feed, daily observations on feeding, behavioural changes, external changes and mortality and biomass determinations are available in our earlier reports [10,11]. Healthy and normal behaviour included belligerent feeding, reaching to the surface while eating and dispersal throughout the water column.

### 2.5. Tissue and Blood Collection

From each tank, four fish were arbitrarily netted out on days 0 and 10 of FD and days 10, 20, 30 and 43 PFD. Two fish were anaesthetized using clove oil [89% eugenol] at 20 μL L^−1^ water [14], followed by a collection of blood via caudal vein puncture [15] using a 2 mL sterile plastic syringe. The non-heparinized blood syringes were kept in a slanting position to facilitate clotting and then incubated at 4 °C overnight. The serum was collected by centrifugation at 1000× *g* for 15 min, transferred to Eppendorf tubes and stored at −20 °C. The remaining two fish, after euthanizing with clove oil (100 μL L^−1^ water) [14,16], were dissected and their entrails removed and thoroughly cleaned. The liver and kidney tissues were collected carefully. For each group of the replicate tank, the respective tissues were pooled, placed in Ziplock bags, labelled and kept at −20 °C. For measuring the oxidative stress parameters, 25 mg of each of the liver and kidney tissues was stored separately, and the remaining amounts were stored for liquid chromatography with tandem mass spectrometry (LC-MS/MS).

### 2.6. Oxidative Stress

Standard kit protocols for the preparation of tissue homogenates for thiobarbituric acid reactive substances (TBARS) as malondialdehyde (MDA), ferric reducing antioxidant power (FRAP), total nitric oxide (TNO) levels and glutathione S-transferase (GST) activity were described in our earlier work [11]. The analyses were done using commercial kits (HiMedia, Mumbai, India), followed by measurement using a UV-vis-spectrophotometer (LabIndia Analytical, Mumbai, India; Model: UV 3200) for GST and TNO and an ELISA reader (Dynamica, Clayton, Australia: Model MPR96) for MDA and FRAP. The GST activity and TNO levels were measured at 340 nm and 540 nm, respectively. The measurements of FRAP and MDA levels were performed at 560 nm and 535 nm, respectively.

### 2.7. Serum Biochemistry

Using commercially available kits and standard protocols, the serum glucose, alanine aminotransferase (ALT) and alkaline phosphatase (ALP), creatinine, calcium and chloride were measured as described in our earlier work [12] using a Photometer (Model: 5010 v5+, Robert Riele KG, Berlin, Germany). The test kits used were glucose, GOD FS 10′ (Diasys Diagnostic Systems, Holzheim, Germany), creatinine, Modified Jaffe’s reaction, Initial rate assay (Span Diagnostics Ltd., Surat, India), ERBA SGPT, IFCC method, Kinetic (Erba Manheim, Manheim, Germany), alkaline phosphatase, FS IFCC 37 °C (Diasys Diagnostic Systems, Holzheim, Germany), calcium, AS FS (Diasys Diagnostic Systems, Holzheim, Germany) and chloride, 21 FS.

### 2.8. Accrual and Depletion of Residues

At the Indian Council of Agricultural Research—Central Institute of Fisheries Technology, Kochi, India, the extraction and purification of FFC and florfenicol amine (FFA) residues were carried out, according to the standard technique with a few minor alterations. The methods for preparing stock and working solutions of FFC and FFA, as well as the extraction of residues, FFC and FFA validation, construction of calibration curves, chromatographic separation using triple quadrupole mass spectrometer AB Sciex 4000 QTRAP and an Exion LC system and data analysis, are outlined vividly in our earlier work [11]. The entire process was validated for selectivity, sensitivity, accuracy, precision, recovery and robustness [11].

### 2.9. Histopathology

The kidney and liver tissues were sampled on days 0 and 10 FD and day 43 PFD from all groups and placed in Bouin’s solution for 1 day for fixation. For tissue preparation, embedding, sectioning (5 μm), and hematoxylin and eosin staining, standard protocols were followed [15]. An advanced trinocular research microscope (Olympus, Tokyo, Japan, Model: BX51) equipped with a 16 MP SCO-LUX camera was used for photomicrography. The captured digital images were edited using ToupTek ToupView software (Version x64, 4.11; Hangzhou ToupTek Photonics Co., Ltd., Hangzhou, China). Histopathological changes were qualitatively evaluated on a six-point ordinal scale as per Bowker et al. [17], in comparison to the control.

### 2.10. Statistical Analysis

The results are presented as a mean ± standard deviation. One-way ANOVA was used to analyze the data on oxidative stress, serum biomarkers and FFC residues. The Tukey post-hoc test was then used to compare means. The Kruskal–Wallis test evaluated the significance of histopathological qualitative scores. Statistical Package tools of version 22.0 for Social Sciences (IBM-SPSS, Armonk, NY, USA) were used for all statistical analyses, with a probability level of *p* < 0.05.

## 3. Results

### 3.1. Mortalities, Feeding Behaviour and Feed Intake upon Florfenicol (FFC)-Dosing

**Figure 1:**
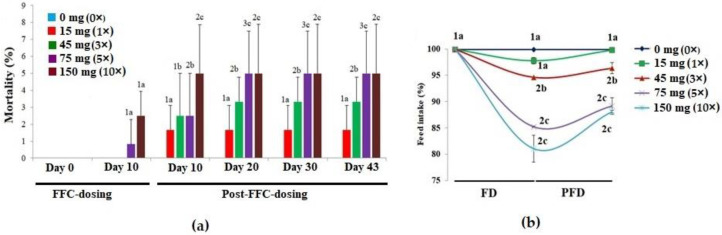
The effects of oral florfenicol (FFC) dosing at 0–10 times the therapeutic dose of 15 mg kg biomass^−1^ day^−1^ for 10 consecutive days on the (**a**) mortality and (**b**) feed intake of *Oreochromis niloticus* juveniles. a–c: Bars of a particular day showing common alphabets differed insignificantly (*p* > 0.05). 1–3: Bars of a particular treatment (dose) showing common numerals differed insignificantly (*p* > 0.05). FD: FFC-dosing; PFD: Post-FFC-dosing.

There were no mortalities during the pre-dosing period (Figure 1a). On day 10 of FD, mortalities were noted only in the 5× and 10× groups, ranging from 0.83 to 2.50%. However, in 10 days of PFD, the 1× group recorded 1.67 ± 1.44% mortalities. A dose-dependent increase in mortalities was noted in the 3×–10× groups (*p* < 0.05) until day 20 PFD. Aggressive feeding was observed in the control and 1× groups. With time, the fish of the 3× and 5× groups became less voracious feeders. The 10× group displayed an increased frequency of suppressed behavioural responses, such as reclining at the bottom of the tank and no interest in feeding during the FD and early PFD periods. The feed intake was in the range of 97.84 ± 0.61% (1×)–81.15 ± 2.53% (10×) of the feed offered on day 10, and the differences among the groups were significant (*p* < 0.05) (Figure 1b). The early PFD phase also showed a reclined feed intake. The 5× and 10× groups excreted more, though there was no difference in the colour of the intestine or excreta upon necropsy. Another striking observation was the shorter time taken for the dosed fish to sedate during the anaesthetizing process.

### 3.2. Abnormalities in Florfenicol (FFC)-Dosed Oreochromis niloticus

A detailed description of the abnormalities in the internal organs of *O. niloticus* juveniles upon necropsy in FD tenure is listed in Table 1. Excessive mucus secretion on the skin and gills was witnessed in the higher-dosed groups (3×–10×). The 10× group often reclined on the tank bottom and showed no curiosity in feed. Most of the fish of the 3×–10× groups exhibited hepatomegaly, splenomegaly and watery kidney during the dosing regimen, which subsided during the PFD period.

### 3.3. LC-MS/MS Analysis

**Figure 2:**
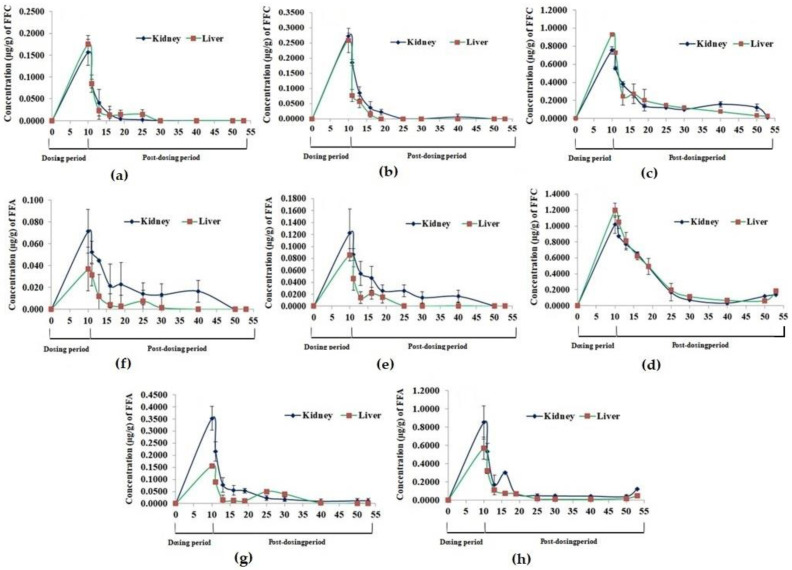
Florfenicol (FFC) and florfenicol amine (FFA) accumulation and depletion profile in *Oreochromis niloticus* juveniles’ kidney and liver tissues upon dietary FFC administration at 0–10 times the therapeutic dose of 15 mg kg biomass^−1^ day^−1^ for 10 consecutive days. (**a**,**e**) 15 mg FFC; (**b**,**f**) 45 mg FFC; (**c**,**g**) 75 mg FFC; (**d**,**h**) 150 mg FFC.

The residues of FFC and FFA were observed to peak significantly (*p* < 0.05) on day 10 of FD in the kidney and liver tissues and reduced significantly (*p* < 0.05) thereafter upon cessation of dosing in all the groups (Figure 2). It took 9 and 20 days for the FFC residues to reach a below-detectable level in the kidney and liver tissues of the 1× group, respectively (Figure 2a). In the 3× group, no FFC residues were detected in the kidney and liver tissues on day 15 and 30 PFD, respectively (Figure 2b). In the higher-dosed groups (5× and 10×), the FFC residues persevered for 43 days or more (Figure 2c,d). The major metabolite of FFC, florfenicol amine (FFA) was also detected in the liver and kidney tissues, although in lesser amounts, compared to its parent compound. The FFA residues in the 1× kidney and liver tissues persevered till 30 and 20 days, respectively, upon cessation of dosing (Figure 2e). Similarly, in the 3× group, the residues were detected till 40 and 30 days of PFD in the kidney and liver tissues, respectively (Figure 2f). The 5× (Figure 2g) and 10× (Figure 2h) kidney and liver tissues showed persistence of FFA residues till day 43 PFD.

### 3.4. Oxidative Stress

#### 3.4.1. Thiobarbituric Acid Reactive Substances (TBARS) as Malondialdehyde (MDA)

**Figure 3:**
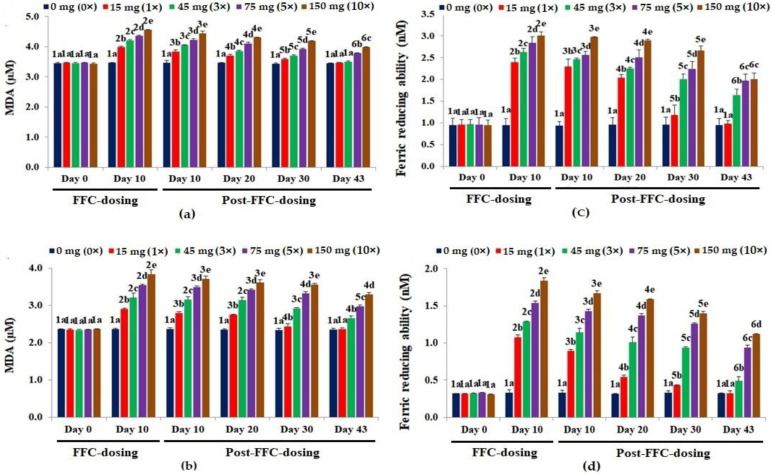
Malondialdehyde [MDA] levels in the (**a**) liver and (**b**) kidney tissues and ferric reducing antioxidant potential [FRAP] in the (**c**) liver and (**d**) kidney tissues of *Oreochromis niloticus* juveniles after dietary florfenicol (FFC) administration at 1–10 times the therapeutic dose of 15 mg kg biomass^−1^ day^−1^ for 10 consecutive days. a–e: Bars of a particular day showing common alphabets differed insignificantly (*p* > 0.05). 1–6: Bars of a particular treatment (dose) showing common numerals differed insignificantly (*p* > 0.05).

A dose-dependent increase (*p* < 0.05) in MDA levels was observed in the liver (Figure 3a) and kidney (Figure 3b) tissues, with the highest levels on day 10 of FD. With the suspension of dosing, the MDA levels of the 1× group recovered fully on day 43 PFD. The kidney MDA levels of the 3× and 5× groups showed an insignificant decrease on day 10 PFD. The recorded MDA levels in the kidney tissues of the 10× group were insignificant on days 10, 20 and 30 PFD.

#### 3.4.2. Ferric-Reducing Antioxidant Power (FRAP)

The 10 days of FD recorded a significant rise in FRAP values (*p* < 0.05) in the liver tissues of all dosing groups (Figure 3c). A dose-dependent increase in FRAP values (*p* < 0.05) in the kidney tissues was observed on day 10 of FD (Figure 3d). The FRAP values in the liver and kidney tissues of the 1× group recovered on day 43 PFD. Although the 3× FRAP values dropped significantly on day 43 PFD, they were still significantly higher than the control (*p* < 0.05), hence also in the 5× and 10× groups.

#### 3.4.3. Total Nitric Oxide (TNO)

**Figure 4:**
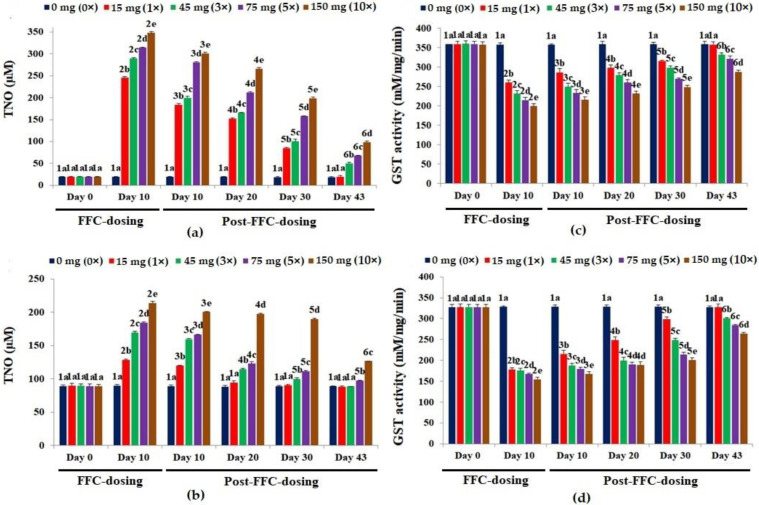
Levels of total nitric oxide [TNO] in the (**a**) liver and (**b**) kidney tissues and glutathione-S-transferase [GST] activity in the (**c**) liver and (**d**) kidney tissues of *Oreochromis niloticus* juveniles after dietary florfenicol (FFC) administration at 1–10 times the therapeutic dose of 15 mg kg biomass^−1^ day^−1^ for 10 consecutive days. a–e: Bars of a particular day showing common alphabets differed insignificantly (*p* > 0.05). 1–6: Bars of a particular treatment (dose) showing common numerals differed insignificantly (*p* > 0.05).

The TNO levels of the liver peaked significantly on day 10 of FD (*p* < 0.05), compared to the control (Figure 4a). Upon cessation of dosing, only the 1× group recouped within 43 days. Alike, a dose-dependent significant hike in TNO levels was observed on day 10 of FD in the kidney (*p* < 0.05), compared to the control (Figure 4b). Within 20 and 43 days of suspension of dosing, the TNO levels in the kidney tissues of the 1× and 3× groups, respectively, reached normalcy. The TNO levels of the 5× and 10× groups were still significantly high on day 43 PFD (*p* < 0.05).

#### 3.4.4. Glutathione-S-Transferase (GST) Activity

The GST activities reduced significantly and dose-dependently (*p* < 0.05) in the liver on day 10 of FD, compared to the control (Figure 4c). The GST activity of the 1× group recovered on day 43 PFD. In the kidney, the FD reduced the GST activities (Figure 4d) significantly (*p* < 0.05). The GST activities of the 1× and 3× groups differed significantly (*p* < 0.05), although the values were quite similar, compared to the other groups. Full recovery was seen only in the 1× group on day 43 PFD.

### 3.5. Serum Biochemistry

**Figure 5:**
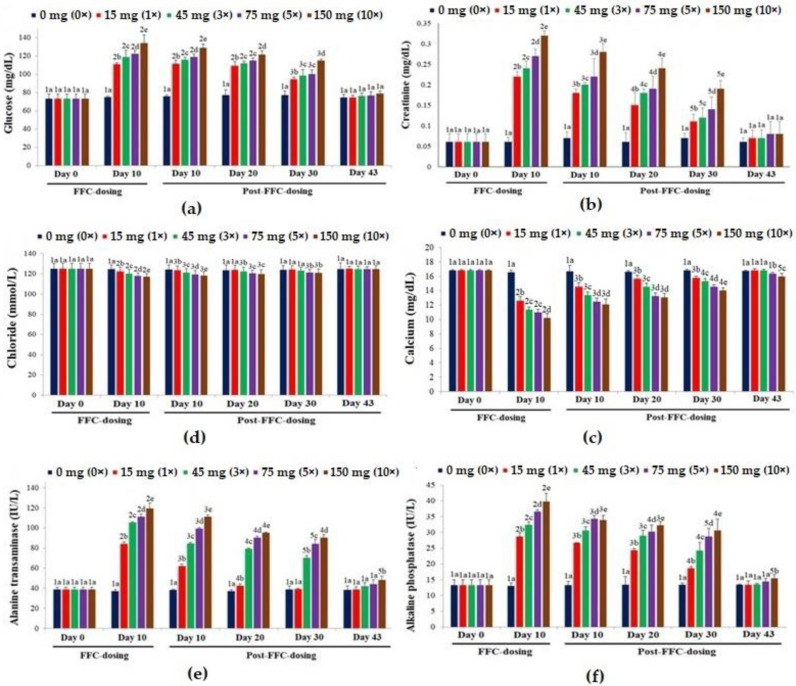
The effects of dietary florfenicol (FFC) administration at 1–10 times the therapeutic dose of 15 mg kg biomass^−1^ day^−1^ for 10 consecutive days on the serum (**a**) glucose, (**b**) creatinine, (**c**) calcium, (**d**) chloride, (**e**) alanine transaminase [ALT] and (**f**) alkaline phosphatase [ALP] levels of *Oreochromis niloticus* juveniles. a–e: Bars of a particular day showing common alphabets differed insignificantly (*p* > 0.05). 1–5: Bars of a particular treatment (dose) showing common numerals differed insignificantly (*p* > 0.05).

The biomarkers of secondary stress (serum glucose [Figure 5a]), kidney function (serum creatinine [Figure 5b]), ionic balance (serum calcium [Figure 5c] and chloride [Figure 5d]) and liver function (serum ALT [Figure 5e] and ALP [Figure 5f]) showed dose-dependent alterations upon dietary FFC administration. Except for the biomarkers of ionic balance, other markers increased significantly (*p* < 0.05). The glucose levels were recouped on day 43 PFD in all the groups. The creatinine levels almost recovered on day 43 PFD, but the levels in comparison with the control were insignificantly high (*p* > 0.05). The calcium levels of the 1× and 3× groups recouped. In the 5× and 10× groups, its levels were still significantly lower than the control on day 43 PFD (*p* < 0.05). The chloride levels of the 1× and 3× groups recouped, respectively, on day 20 and day 30 PFD, while those of the 5× and 10× groups normalized at the end of the experimental tenure. The ALT levels of the 1× group were recouped on day 30 PFD, whereas the 3× and 5× groups recovered on day 43 PFD. Although all the groups showed ALP levels within the control range, the 10× group showed significantly higher values (*p* < 0.05) on day 43 PFD.

### 3.6. Kidney and Liver Histopathology

**Figure 6:**
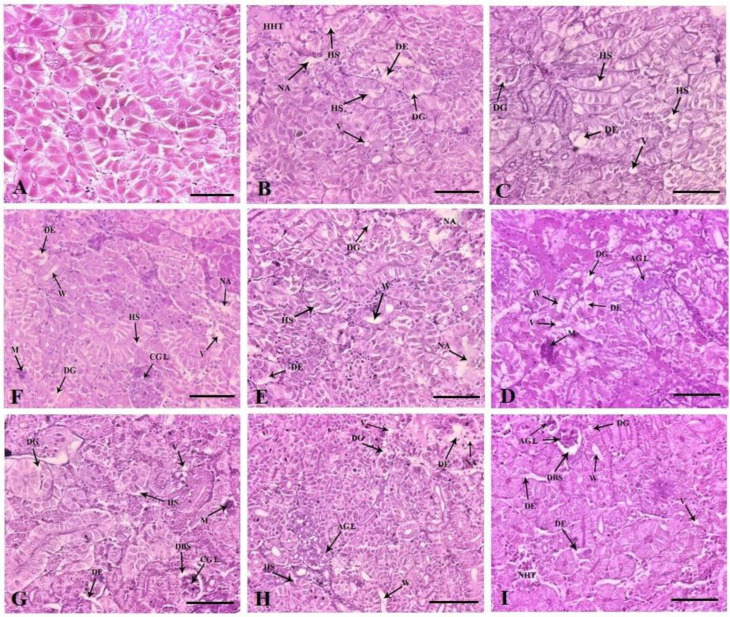
Histoarchitectural structures of the kidney tissues of (**A**) control with normal tissue architecture ×200 and dietary florfenicol (FFC)-administered *Oreochromis niloticus* juveniles for 10 consecutive days on day 10 FFC-dosing at (**B**) 15 mg kg biomass^−1^ day^−1^ [1×], (**C**) 45 mg kg biomass^−1^ day^−1^ [3×], (**D**) 75 mg kg biomass^−1^ day^−1^ [5×] and (**E**) 150 mg kg biomass^−1^ day^−1^ [10×] ×200, and on day 43 post FFC dosing at (**F**) 1×, (**G**) 3×, (**H**) 5× and (**I**) 10×, ×200 showing degeneration of renal tubules (DG) and tubular epithelium (DE), hydropic swelling (HS), necrotized areas (NA), external vacuolation (V), hypoplastic haematopoietic tissues (HHT), mineralization (M), widening of the lumen (W), atrophic glomerulus (AGL), collapsed glomerulus (CGL), necrotised haematopoietic tissues (NHT) and dilated Bowman’s space (DBS). H&E staining, Scale Bars (**A**–**I**): 25 μm.

The control fish kidney displayed a normal histoarchitecture (Figure 6A). Dietary FFC administration for 10 days caused marked tubular epithelial and renal tubular degeneration in the 1× group (Figure 6B). Alterations such as hydropic swelling, hypoplastic haematopoietic areas and vacuolation were documented. The 3× group demonstrated similar changes, with higher vehemence (Figure 6C). In the 5× group, the mineralization increased considerably, with the prominence of the widened lumen and atrophied glomerulus (Figure 6D). The 10× group showed severe tubular epithelial and renal tubular degeneration, widened lumen, hydropic swelling and severely necrotized areas (Figure 6E). The qualitative scores of the histoarchitectural changes are portrayed in Table 2. The tubular epithelial and renal tubular degeneration were the most obvious change observed in the FFC-dosed juveniles. With the cessation of dosing, a reduction in kidney histopathological alterations was observed. The 1× (Figure 6F) and 3× (Figure 6G) groups, however, showed mild persistence of aberrations. The 5× group showed the perseverance of necrotized areas, renal tubular degeneration and widened lumen (Figure 6H). The 10× group showed a meagre recovery, with collapsed glomerulus, dilated Bowman’s space, necrotized haematopoietic tissues, degenerative renal tubules and tubular epithelium (Figure 6I and Table 2).

**Figure 7:**
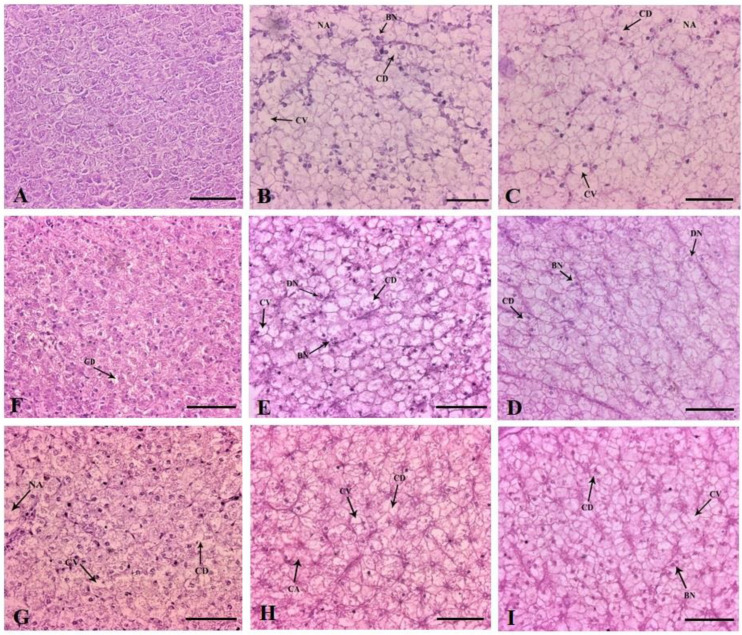
Histoarchitectural structures of the liver tissues of (**A**) control with normal hepatocytes and parenchyma ×200 and dietary florfenicol (FFC)-administered *Oreochromis niloticus* juveniles for 10 consecutive days on day 10 FFC-dosing at (**B**) 15 mg kg biomass^−1^ day^−1^ [1×], (**C**) 45 mg kg biomass^−1^ day^−1^ [3×], (**D**) 75 mg kg biomass^−1^ day^−1^ [5×] and (**E**) 150 mg kg biomass^−1^ day^−1^ [10×], ×200, and on day 43 post FFC dosing at (**F**) 1×, (**G**) 3×, (**H**) 5× and (**I**) 10×, ×200 showing glycogen-type vacuolation, cytoplasmic vacuolation (CV), cytoplasmic degeneration (CD), necrotised areas (NA) and binucleated cells (BN); H&E staining. Scale Bars (**A**–**I**): 25 μm.

Histologically, the liver of the control fish was characterized by normal parenchyma and regular hepatocytes (Figure 7A). The 1× group had glycogen-type vacuolation (severe), cytoplasmic vacuolation (mild), cytoplasmic degeneration and necrotized areas on day 10 of FD. The hepatocyte nuclei were generally aggregated towards the hepatic cords (Figure 7B). Similar changes were observed in an increased fashion, correlating with dose increment (Figure 7C–E). Nuclear disorders, such as binucleated hepatocytes and degenerative hepatocytes, were profoundly visible in all the dosing groups. In the therapeutic group, the recovery of the liver tissue architecture was demonstrated on the last day of experimentation. The glycogen-type vacuolation was significantly reduced in the 1× group (Figure 7F). The 3×–10× groups disclosed no recoveries with the persistence of nuclear anomalies (Figure 7G–I and Table 2).

## 4. Discussion

As several drug-induced organ toxicities negatively impacted fish welfare and food safety [2], this study aimed to identify, evaluate and distinguish the potential renal toxicity and hepatotoxicity of FFC at 0–10 times its maximum therapeutic dose when orally administered for 10 days to guide its responsible use in tropical aquaculture. As the most influential organs in fish physiology, the kidney and liver are deemed the main targets of drug-induced toxicity [10,11]. Up to now, there were sparse reports on the negative effects of FFC on the fish kidney and liver tissues in tropical conditions. In this study, the feed intake abated significantly in a dose-dependent fashion during the dosing tenure, hinting at the reduced acceptability of FFC feed. The higher-dosed groups produced mortalities during the dosing tenure, which proclaimed the dietary FFC-induced toxicity. The mortalities in the 1× group increased during the PFD period, hinting at the perseverance of stress and the breach of the tolerance level of the fish. In similar experiments, no mortalities were observed during the dosing period at the therapeutic or lower FFC dose levels [11,18]. Florfenicol is known to cause diarrhoeic conditions in mice [19], although the mechanism behind this is still unknown. An excess faecal secretion was observed in the current study in higher FFC-dosed *O. niloticus* juveniles, possibly due to the irritable and bactericidal properties of FFC, thus causing the release of endotoxins or microbial dysbiosis [20] during therapy. Further, the far quicker sedation of FFC-dosed *O. niloticus* than the control indicated the stress the fish endured, possibly due to the inhibition of hepatic cytochrome P450 [21] by the FFC and FFA residues. The LC-MS/MS analysis demonstrated good recoveries between 91.64 and 93.84% of FFC in spiked muscle. In addition, the perseverance of FFA residues was longer and higher at the higher doses (3×–10×), justifying the positive relationship between increased drug doses and the perseverance of their corresponding metabolites. Yang et al. [22] also observed similar perseverance of FFA residues in crucian carp, compared to its parent molecule FFC, which was eliminated faster. However, the quicker elimination of FFC can also be attributed to faster metabolism in tropical climates [22].

The significant increase in the levels of glucose indirectly indicated the release of stress hormones, such as cortisol and epinephrine, upon FFC-induced stress [23]. FFC is known to be hepatotoxic and toxic to renal tissues [11,24], hence, the elevated levels of serum ALT, ALP and creatinine. The hike in the liver and kidney tissue FFC and FFA residues and their possible interaction with the cell membranes further justified these observations. As FFA has a high half-life in the liver and kidney, the persistence of the metabolite in the higher-dosed groups was imminent [25]. The present study’s considerable decrease in calcium and chloride levels induced changes in membrane permeability that were either caused by a decrease in ionic compound intake into the body or by impaired inorganic-ionic compound uptake processes at the gill [26]. According to Wei et al. [27], FFC is known to alter cell permeability, which could impact the release and uptake of blood plasma ions. As a result, *O. niloticus* might have developed an imbalance in intracellular and extracellular fluids or diminished outward electrogenic proton pumping and a slower rate of cation and anion entry, leading to hypochloremia and hypocalcemia [26]. Additionally, it has been claimed that antibiotics, such as aminoglycosides, activate the chloride channels in the renal tubules, causing an excessive loss of chloride [28,29]. It is known that the metabolic byproducts of some antibiotics, such as tetracyclines, accumulate inside the mitochondria of renal cells and prevent oxidative phosphorylation, leading to metabolic acidosis [29]. Acidosis is often concomitant with reduced plasma calcium and chloride levels [30]. The histopathological observations of the current study further confirmed the cellular damage. The excessive faecal secretion might have also led to an ionic imbalance, leading to a fall in serum calcium and chloride levels [31].

The inability to produce an antioxidant defence system leads to the expression of oxidative stress [5]. The earlier studies asserted the ability of FFC to inflict oxidative stress on *O. niloticus* juveniles [5,11]. Oxidative stress participates in the liver fibrogenic response and often results in fibrosis. As the watery kidney was observed upon necropsy, the organ damage due to oxidative stress can be vindicated. The kidney has a high capacity for the uptake of lipid-binding proteins and lipid-regulating hormones [32], and peroxidation of lipids persists in the kidney tissues. As renal injury upon oxidative stress has been documented in several animals [5,32], the documentation of glomerulopathy and degeneration of renal tubules upon dietary FFC administration in *O. niloticus* was considered befitting. The observations on the increase in MDA levels and decreased GST activity due to the drug sulfasalazine in rat liver and kidney tissues were concomitant with the present study, implying considerable hepatic and renal toxicity [33]. They also reported degeneration of renal tubules, cellular injury, depolymerization of polysaccharides and liver parenchyma fragmentation upon oral administration of the drug, obviously linking it with the symptoms of oxidative stress. The concomitant increase in the ROS levels within these tissues upon FFC administration led to the oxidation of polyunsaturated fatty acids (PUFA) and peroxidation of lipid/glycogen molecules, profoundly available in these tissues [34]. Lipid peroxidation is also caused by events such as disruptions in electron transport, decoupling of oxidative phosphorylation, increased mitochondrial respiration and increased oxygen consumption [2]. Substances, such as NADPH-cytochrome P450 in the reductase-dependent processes in microsomes, are thought to play a significant role in FFC metabolism, and can also cause lipid peroxidation [34].

The physiology of FFC in fish liver and kidney is sparsely documented. The FFC and FFA might have hampered the homeostatic effects of antioxidants and ROS, leading to an increase in the oxidation of PUFAs. Undoubtedly, it resulted in a hike in MDA levels, similar to Chatterjee et al. [35], leading to hepatomegaly and watery kidneys. It is unclear whether MDA directly harms cells, or if the rate of lipid peroxidation reactions simply rose when oxygen radicals outnumbered antioxidants. Nevertheless, as evinced by the higher MDA levels and histopathological alterations of the present study, the cells that collected lipid peroxidation byproducts were less likely to function properly. The increased levels of MDA in the liver and kidney tissues of *O. niloticus* destroyed the antioxidant enzymes and their substrates, thus reducing the antioxidant capacity [5,18,36]. Further, with the suspension of dosing, the MDA levels of the lower-dosed (1×–3×) groups recouped in the liver tissues. However, the 3×–10× groups showed considerably high MDA values in the kidney tissues on day 43 PFD, implying extensive kidney damage.

The reduction in GST activity was significant and dose-dependent on day 10 in the liver and kidney tissues, as in previous studies on FFC [5] and other pharmaceuticals [37]. Under normal physiological conditions, the liver is protected from oxidative stress by the capacity of its hepatocytes to synthesize glutathione (GSH), which plays a pivotal role in the functioning of mitochondria, where oxygen consumption and generation of ROS occur [38]. FFC is known to be an inhibitor of mitochondrial protein synthesis, which can induce noticeable cytotoxicity and, thus, reduce the re-synthesis of intracellular GSH levels. Oxidative injury and fibrosis are proclaimed to be the leading cause of GSH depletion [39]. In our study, the decline in GST activity may be linked to the short-term decrease of intracellular GSH in the liver and kidney tissues that were exposed to FFC. Equally, FFA also inhibited intracellular GSH accumulation. Therefore, disruption of intracellular redox homeostasis via the inhibition of GSH and the accumulation of ROS may magnify oxidative stress to affect the tissues, causing considerable renal toxicity and hepatotoxicity.

The substantial increase in the FRAP values in the FFC-dosed groups proclaimed the intensity of oxidative tissue damage. Together with the considerable increase in MDA levels, i.e., lipid peroxidation, which is generally accompanied by the production of ROS and free radicals, the vehemence of oxidative stress is imminent. Further, the reduction of GST activity correlated with the aberrations caused by ROS in the liver and kidney tissues. Previous research on FRAP of enzymatic proteolysis concluded that the reducing power activity was related to several variables, including peptide size and molecular weight, amino acid sequence, number of hydrophobic amino acids and quantity of sulphur-containing and acidic amino acids [40]. Similarly, a hike in FRAP values was documented upon oxidative stress in *C. carpio* due to exposure to copper [41], terbuthylazine [42] and cadmium-treated mice [43]. The recovery was, however, faster in the liver tissues due to the ameliorative effect of liver tissues, considering FFC toxicity [43].

Significantly high TNO levels were documented in the vital organs upon FD. Nitric oxide (NO) is produced by a reaction that is catalyzed by NO synthases (NOS), which are available in three isoforms, all of which are profoundly available in the kidney [44]. The documentation of lower TNO levels in the healthy fish liver than in the kidney tissues was, thus, imminent [11,45]. However, the rate of increase in TNO levels in the liver tissues was quite higher. It is justified that FFC vivified NO production, both in the kidney and liver tissues, thus proclaiming its substantial role in the development of oxidative stress in *O. niloticus*, similar to zebrafish *Danio rerio* [44]. The increased concentration of TNO could probably be one of the main reasons for hepatomegaly, as observed upon necropsy. Possibly, the oral administration of FFC enhanced the activity of NOS, leading to the overproduction of NO in the target tissues. Iwakiri and Kim [46] concluded that inducible NOS (iNOS)-derived TNO is deleterious because it mainly acts as a pro-inflammatory mediator. Perhaps the increased NO levels of the current study at the higher FFC-dosed groups had an inimical effect on fish liver tissues, as confirmed by the histopathological findings and serum ALP levels.

Kidneys are particularly vulnerable to toxic injury because they are exposed to blood plasma [4]. FFC is also known to cause cell cycle arrest and to inhibit the proliferation of haematopoietic cells and apoptosis [46]. Mineralization can appear in any segment of the renal tubule and is often associated with calcium and phosphorus ratio discrepancy in the diet [47]. The triggering events of mineralization are not yet clearly defined in fish, although it is likely that an initial cytotoxic insult to the cellar components of the renal tubules can initiate the process [48]. The induction of mineralization, even at the therapeutic dietary FFC dose in *O. niloticus* [11], indicated the toxic potential of FFC towards renal tissues. The observations on the marked degeneration of the renal tubules in FFC-dosed fish, similar to Gaikowski et al. [7], portrayed that the damage may be toxic. The persistence of renal tubular degeneration at a moderate level even on day 43 PFD implied that the FFC-induced toxicity at the therapeutic dose may lead to tissue necrosis [46]. The documentation of glomerulopathy, with dilated Bowman’s space, during the FD and PFD periods in the 5× and 10× groups suggested renal toxicity, tissue damage and reduction in excretion rates [7]. The widened lumen of the 1× group is suggestive of excessive elimination rates. The manifestation of the renal tubule hydropic swelling with intact nuclei supported the results of Gaikowski et al. [7]. The shrunk lumen and constricted tubules observed in the higher-dosed groups are suggestive of stalled tubular reabsorption [7]. After metabolic conversion to FFA, FFC is virtually entirely removed by the kidneys, presumably through glomerular filtration [49]. The fish kidney is probably the organ that has been examined the most. Yet, there is still no agreement on the mechanism that causes damage to tubular cells and how acute renal insufficiency is triggered. However, it can be hypothesized that following administration and metabolism, FFA is passed in the glomerular filtrate and is transported into the tubular cells of the nephron. Within the tubular cells, high FFA concentrations occur, which interferes with several important metabolic pathways, such as protein reabsorption, protein synthesis, mitochondrial respiration and the sodium-potassium pump, as amphenicols are known to interfere with mitochondrial functioning [47].

In the current study, necrosis was confined to the interstitial tissues, whereas degeneration was observed profoundly in the renal tubules, where absorption of the drug occurs. The degeneration of tubules may result in lysosomal leakage from the tubular cells due to oxidative damage [6]. As a result, there may be an increase in the risk of renal toxicity, renal vasoconstriction, decreased renal blood flow, decreased glomerular filtration, and an increase in peak and trough FFA levels. The above statement was supported by the persistence of FFA residues in the renal tissues of the present study. FFC is lipophilic, which makes it appropriate for inducing oxidative type (lipid peroxidation) and ionophoric damage [7]. This latter type of damage is triggered by the development of pores or channels in the brush border, which results in the leaking of cell content and an increase in the intracellular uptake of tiny solutes, which are then deposited inside the renal tissues, increasing mineralization. The reduction in serum cations brought about by mineralization was accompanied by a reduction in calcium after FFC treatment. When the dosage was stopped, the 1× group’s lumen widened, suggesting difficulty with filtrate flow and poor glomerulus function, potentially by the persistence of FFA residues.

The observed glycogen-type vacuolation on day 10 of FD indicated the utilization of glycogen reserves [50] and the severe hepatotoxic potential of FFC. The extensive cellular vacuolation, as indicated by increased liver cell space, blurred cell boundaries and liver cell fatty degeneration, was profoundly recorded in FFC-dosed groups, similar to an earlier study [2]. Normally, only a minor volume of ALT and ALP are released into the blood. The acutely damaged liver tissues or increased cell membrane permeability of FFC-dosed *O. niloticus* caused a significant increase in serum ALT and ALP levels. The weakened GST activity allowed the production of too many free radicals to attack PUFA, leading to a hike in MDA levels, a manifestation of oxidative damage, which destroy the functional integrity of cells. The apparent liver damage caused by FFC was possibly due to the accelerated metabolism of drugs and the production of FFA, which persevered considerably, compared to the parent compound, and increased oxidative stress damage in the liver tissues.

Increased hepatocyte vacuolation, possibly linked to the FFC dose, is an indication of a degenerative process [7] that may cause metabolic injury, alterations to the integrity of cell membranes and ion pump degradation [2]. The intense vacuolation caused the clustering of nuclei in the direction of the hepatic sinusoids, a prominent nuclear abnormality. The gathering of hepatocyte nuclei towards the sinusoids, which allows for blood flow and filtration [51], may have enhanced cellular detoxification and oxygen uptake of the hepatocytes. The demonstration of the presence of degenerating nuclei and hepatocytes devoid of nuclei indicated that the FFC-induced hepatotoxicity was severe. In contrast, the liver tissues of FFC-dosed fish in previous studies lacked any abnormalities [52,53]. The biochemical findings further backed up the evidence of the liver and kidney histopathological findings. As hepatocytes undergo rigorous metabolism and detoxification, they are frequently bi-nucleated [51]. Though the mitotic binucleate hepatocytes were found in the current investigation, the numbers are not plenty. The liver’s inability to regenerate was, thus, supported by the lack of excessive numbers of mitotic cells. Nevertheless, the recovery of hepatocytes led to an increase in nuclear numbers, similar to an earlier study [50]. The loss of membrane structure may cause the discharge of cellular components into the extracellular environment, as evidenced by the oxidative stress data of the current investigation. Despite its primary mode of action on the inhibition of protein synthesis on bacterial ribosomes, FFC has been shown to inhibit a variety of other cellular processes, including ATPases [5], compromising its normal functioning in gills [23] and, thus, threatening ionic homeostasis, enzymatic activity and cell membrane integrity [4].

## 5. Conclusions

Overall, our results indicated an accrual of FFC and FFA residues in the kidney and liver tissues following FFC administration, which significantly altered their physiology. Histopathological examination of kidney and liver tissues indicated that the therapeutic (1×) and the higher doses of FFC (3×–10×) can significantly damage these organs, leading to structural disorders at the tissue level. Nevertheless, the FFC exhibited severe nephrotoxicity and hepatotoxicity at higher doses, which reminded us to pay great attention to its side effects. It can be inferred that FFC induces reversible renal toxicity and hepatotoxicity and is well tolerated by *O. niloticus* juveniles. The associated changes and behavioural anomalies in the therapeutic group are, however, considered normal. Our study is probably the first to provide the experimental basis for reasonably applying FFC in aquaculture under tropical conditions, which is of great importance for securing fish welfare, food safety and human health.

## Figures and Tables

**Table 1:** Recorded abnormalities in *Oreochromis niloticus* juveniles after dietary florfenicol (FFC) administration at 1–10 times the therapeutic dose of 15 mg kg biomass^−1^ day^−1^ for 10 consecutive days in comparison to control.

Groups	Experimental Period	Liver	Kidney	Gills	Spleen	Peritoneum	Skin
3×	FD	Hepatomegaly	NC	NC	Splenomegaly	NC	Mucous secretion
	PFD	Hepatomegaly (30)	NC	NC	NC	NC	NC
5×	FD	Hepatomegaly	Watery	NC	Splenomegaly	NC	Mucous secretion
	PFD	Hepatomegaly (30)	NC	Mucous secretion (20)	Splenomegaly (10)	NC	Mucous secretion (10)
10×	FD	Hepatomegaly	Watery	NC	Splenomegaly	Blackish	Mucous secretion
	PFD	Hepatomegaly (30)	NC	Mucous secretion (20)	Splenomegaly (10)	NC	Mucous secretion (10)

The 1× group showed no changes or alterations during the experimental tenure. NC: No change; FD: FFC-dosing period; PFD: Post-FFC-dosing period. Values in parentheses indicate the days of persistence during the PFD.

**Table 2:** The histoarchitectural changes in *Oreochromis niloticus* juveniles upon dietary FFC administration at 1–10 times the therapeutic dose of 15 mg kg biomass^−1^ day^−1^ for 10 consecutive days as assessed qualitatively on a 6-point ordinal scale * in comparison to the normal architecture.

Organ	1×		3×		5×		10×	
	10FD	43PFD	10FD	43PFD	10FD	43PFD	10FD	43PFD
Kidney								
DG	3.03 ± 0.08 ^1a^	2.12 ± 0.07 ^2b^	3.14 ± 0.04 ^1ad^	2.37 ± 0.11 ^2c^	3.18 ± 0.04 ^1d^	2.43 ± 0.10 ^2c^	3.26 ± 0.04 ^1d^	2.70 ± 0.14 ^2e^
DE	1.38 ± 0.11 ^1a^	0.48 ± 0.24 ^2b^	1.54 ± 0.10 ^1c^	0.97 ± 0.10 ^2d^	1.64 ± 0.11 ^1e^	1.11 ± 0.07 ^2f^	1.74 ± 0.10 ^1e^	1.37 ± 0.08 ^2a^
HS	1.41 ± 0.07 ^1a^	0.63 ± 0.29 ^2b^	1.58 ± 0.14 ^1c^	1.07 ± 0.14 ^2d^	1.64 ± 0.10 ^1c^	1.12 ± 0.07 ^2e^	1.77 ± 0.10 ^1f^	1.75 ± 0.10 ^1f^
V	1.28 ± 0.10 ^1a^	1.03 ± 0.13 ^2b^	1.44 ± 0.10 ^1c^	1.26 ± 0.11 ^2a^	1.58 ± 0.13 ^1d^	1.37 ± 0.11 ^2c^	1.49 ± 0.07 ^1e^	1.40 ± 0.10 ^1e^
NA	1.18 ± 0.11 ^1ac^	0.73 ± 0.21 ^2b^	1.28 ± 0.10 ^1ade^	1.14 ± 0.10 ^2c^	1.31 ± 0.07 ^1def^	1.27 ± 0.11 ^1d^	1.38 ± 0.07 ^1ef^	1.33 ± 0.07 ^1f^
GL	1.09 ± 0.07 ^1ac^	0.43 ± 0.15 ^2b^	1.17 ± 0.10 ^1a^	1.05 ± 0.13 ^2c^	1.18 ± 0.07 ^1a^	1.15 ± 0.10 ^1a^	1.32 ± 0.07 ^1d^	1.29 ± 0.07 ^1d^
M	1.12 ± 0.07 ^1ad^	0.97 ± 0.10 ^2b^	1.21 ± 0.07 ^1ac^	1.11 ± 0.07 ^2d^	1.24 ± 0.07 ^1c^	1.12 ± 0.11 ^2d^	1.38 ± 0.08 ^1e^	1.30 ± 0.07 ^1e^
**Liver**								
GV	4.59 ± 0.05 ^1a^	3.46 ± 0.04 ^2b^	4.67 ± 0.02 ^1c^	3.54 ± 0.04 ^2b^	4.69 ± 0.02 ^1ad^	3.59 ± 0.09 ^2e^	4.73 ± 0.03 ^1d^	4.60 ± 0.05 ^2a^
CV	1.31 ± 0.07 ^1ac^	1.27 ± 0.10 ^1a^	1.42 ± 0.07 ^1bc^	1.28 ± 0.13 ^2c^	1.44 ± 0.10 ^1bc^	1.30 ± 0.14 ^2a^	1.59 ± 0.09 ^1d^	1.39 ± 0.07 ^2c^
CD	1.04 ± 0.14 ^1a^	1.00 ± 0.10 ^1a^	1.11 ± 0.09 ^1a^	1.09 ± 0.07 ^1a^	1.23 ± 0.08 ^1b^	1.17 ± 0.10 ^2b^	1.45 ± 0.10 ^1c^	1.31 ± 0.06 ^2c^

Values are represented as mean ± standard deviation. DG: Degeneration of renal tubule; DE: Degeneration of renal tubular epithelium; HS: Hydropic swelling; V: Vacuolation; NA: Necrotised areas; GL: Glomerulopathy including atrophic glomerulus and collapsed glomerulus and dilated Bowman’s space (DBS); M: mineralization; GV: Glycogen-type vacuolation; CV: cytoplasmic vacuolation; CD: cytoplasmic degeneration. FD: FFC-dosing; PFD: Post-FFC-dosing. Values are the mean of six observations for each organ of the respective group. a–f: Values for a particular row and a particular histopathological change showing common alphabetical superscripts differed insignificantly (*p* > 0.05). 1–2: Values for a particular treatment (dose) and a particular histopathological change showing common numeral superscripts differed insignificantly (*p* > 0.05). * Six-point ordinal scale: 0—No change, 1—Normal; <5% of tissues affected, 2—Mild; 5–15% of tissues affected, 3—Moderate; 15–25% of tissues affected, 4—Marked; 25–50% of tissues affected and 5—Severe; >50% of tissues affected.

## Data Availability

The data presented in this study are available on request from the corresponding author.

## References

[1] Feng J.B., Huang D.R., Zhong M., Liu P., Dong J.D. (2016). Pharmacokinetics of florfenicol and behaviour of its metabolite florfenicol amine in orange-spotted grouper (*Epinephelus coioides*) after oral administration. J. Fish Dis..

[2] Limbu S.M., Chen L.Q., Zhang M.L., Du Z.Y. (2021). A global analysis on the systemic effects of antibiotics in cultured fish and their potential human health risk: A review. Rev. Aquac..

[3] Patil P.K., Mishra S.S., Pradhan P.K., Manna S.K., Abraham T.J., Solanki H.G., Shahi N., Swain P., Sahoo S.N., Avunje S. (2022). Usage pattern of chemicals, biologicals and veterinary medicinal products in Indian aquaculture. Rev. Aquac..

[4] Rodrigues S., Antunes S.C., Nunes B., Correia A.T. (2019). Histopathological effects in gills and liver of *Sparus aurata* following acute and chronic exposures to erythromycin and oxytetracycline. Environ. Sci. Pollut. Res..

[5] Shiroma L.S., Soares M.P., Cardoso I.L., Ishikawa M.M., Jonsson C.M., Queiroz S.C.N. (2020). Evaluation of health and environmental risks for juvenile tilapia (*Oreochromis niloticus*) exposed to florfenicol. Heliyon.

[6] Gaikowski M.P., Wolf J.C., Endris R.G., Gingerich W.H. (2003). Safety of Aquaflor (florfenicol, 50% type A medicated article), administered in feed to channel catfish, *Ictalurus punctatus*. Toxicol. Pathol..

[7] Gaikowski M.P., Wolf J.C., Schleis S.M., Tuomari D., Endris R.G. (2013). Safety of florfenicol administered in feed to tilapia (*Oreochromis* sp.). Toxicol. Pathol..

[8] Gaunt P.S., Gao D., Sun F., Endris R. (2010). Efficacy of florfenicol for control of mortality caused by *Flavobacterium columnare* infection in channel catfish. J. Aquat. Anim. Health.

[9] Jarau M., MacInnes J.I., Lumsden J.S. (2019). Erythromycin and florfenicol treatment of rainbow trout *Oncorhynchus mykiss* (Walbaum) experimentally infected with *Flavobacterium psychrophilum*. J. Fish Dis..

[10] Bardhan A., Abraham T.J., Singha J., Saha S., Sarker S., Patil P.K. (2022). The effects of extended feeding of florfenicol coated medicated diets on the safety, serum biomarkers and blood cells morphology of Nile tilapia *Oreochromis niloticus* (L.). Environ. Sci. Pollut. Res..

[11] Bardhan A., Abraham T.J., Singha J., Sar T.K., Rajisha R., Krishna E.K.N., Kumar K.A., Patil P.K. (2022). Histopathological aberrations and oxidative stress responses in Nile tilapia *Oreochromis niloticus* as influenced by dietary florfenicol and its metabolites. Aquaculture.

[12] Bardhan A., Abraham T.J., Das R., Patil P.K. (2022). Biological Responses of Nile tilapia *Oreochromis niloticus* as influenced by dietary florfenicol. Toxics.

[13] CPCSEA (Committee for the Purpose of Control and Supervision of Experiments on Animals) (2021). Guidelines of CPCSEA for Experimentation on Fishes.

[14] AVMA (2020). AVMA Guidelines for the Euthanasia of Animals: 2020 Edition.

[15] Roberts R.J. (2012). Fish Pathology.

[16] Davis D.J., Klug J., Hankins M., Doerr H.M., Monticelli S.R., Song A., Gillespie C.H., Bryda E.C. (2015). Effects of clove oil as a euthanasia agent on blood collection efficiency and serum cortisol levels in *Danio rerio*. J. Am. Assoc. Lab. Anim. Sci..

[17] Bowker J.D., Carty D., Bowman M.P. (2013). The safety of Aquaflor (50% florfenicol) administered in feed to fingerling yellow perch. N. Am. J. Aquac..

[18] Elia A.C., Pacini N., Fioravanti M.L., Dörr A.J.M., Zaccaroni A., Parmeggiani A.M., Gustinelli A., Mordenti O., Abete M.C., Prearo M. (2016). Assessment of detoxifying markers for florfenicol in rainbow trout liver. J. Aquat. Anim. Health.

[19] Hu D., Han Z., Li C., Lv L., Cheng Z., Liu S. (2016). Florfenicol induces more severe hemotoxicity and immunotoxicity than equal doses of chloramphenicol and thiamphenicol in Kunming mice. Immunopharmacol. Immunotoxicol..

[20] Sumithra T.G., Sharma K.S., Gangadharan S., Suresh G., Prasad V., Amala P.V., Sayooj P., Gop A.P., Anil M.K., Patil P.K. (2022). Dysbiosis and restoration dynamics of the gut microbiome following therapeutic exposure to florfenicol in snubnose pompano (*Trachinotus blochii*) to aid in sustainable aquaculture production strategies. Front. Microbiol..

[21] Saba A.B., Ola-Davies O., Oyeyemi M.O., Ajala O. (2000). The toxic effects of prolonged administration of chloramphenicol on the liver and kidney of rats. Afr. J. Biomed. Res..

[22] Yang F., Yang F., Wang G., Kong T., Wang H., Zhang C. (2020). Effects of water temperature on tissue depletion of florfenicol and its metabolite florfenicol amine in crucian carp (*Carassius auratus gibelio*) following multiple oral doses. Aquaculture.

[23] Biswal A., Srivastava P.P., Krishna G., Paul T., Pal P., Gupta S., Varghese T., Jayant M. (2021). An integrated biomarker approach for explaining the potency of exogenous glucose on transportation induced stress in Labeo rohita fingerlings. Sci. Rep..

[24] Zhang Y., Guo P., Wang M., Wu Y., Sun Y., Su H., Deng J. (2021). Mixture toxicity effects of chloramphenicol, thiamphenicol, florfenicol in Daphnia magna under different temperatures. Ecotoxicology.

[25] Marques T.V., Paschoal J.A.R., Barone R.S.C., Cyrino J.E.P., Rath S. (2018). Depletion study and estimation of withdrawal periods for florfenicol and florfenicol amine in pacu (*Piaractus mesopotamicus*). Aquac. Res..

[26] Sathya A., Prabhu T., Ramalingam S. (2020). Structural, biological and pharmaceutical importance of antibiotic agent chloramphenicol. Heliyon.

[27] Wei C.F., Shien J.H., Chang S.K., Chou C.C. (2016). Florfenicol as a modulator enhancing antimicrobial activity: Example using combination with thiamphenicol against *Pasteurella multocida*. Front. Microbiol..

[28] Remen M., Imsland A.K., Stefansson S.O., Jonassen T.M., Foss A. (2008). Interactive effects of ammonia and oxygen on growth and physiological status of juvenile Atlantic cod (*Gadus morhua*). Aquaculture.

[29] Kelly D.J., Cox A.J., Tolcos M., Cooper M.E., Wilkinson-Berka J.L., Gilbert R.E. (2002). Attenuation of tubular apoptosis by blockade of the renin-angiotensin system in diabetic Ren-2 rats. Kidney Int..

[30] Casado F., Mudunuru S.A., Nasr R. (2018). A case of hypokalemia possibly induced by Nafcillin. Antibiotics.

[31] Collett S.R. (2012). Nutrition and wet litter problems in poultry. Anim. Feed. Sci. Technol..

[32] Sreejai R., Jaya D.S. (2010). Studies on the changes in lipid peroxidation and antioxidants in fishes exposed to hydrogen sulfide. Toxicol. Int..

[33] Linares V., Alonso V., Albina M.L., Bellés M., Sirvent J.J., Domingo J.L., Sánchez D.J. (2009). Lipid peroxidation and antioxidant status in kidney and liver of rats treated with sulfasalazine. Toxicology.

[34] Alessio H.M. (2000). Lipid peroxidation in healthy and diseased models: Influence of different types of exercise. Handbook of Oxidants and Antioxidants in Exercise.

[35] Chatterjee A., Bhattacharya R., Chatterjee S., Saha N.C. (2021). Acute toxicity of organophosphate pesticide profenofos, pyrethroid pesticide λ cyhalothrin and biopesticide azadirachtin and their sublethal effects on growth and oxidative stress enzymes in benthic oligochaete worm, *Tubifex tubifex*. Comp. Biochem. Physiol. C Toxicol. Pharmacol..

[36] Ren X., Wang Z., Gao B., Liu P., Li J. (2017). Effects of florfenicol on the antioxidant status, detoxification system and biomolecule damage in the swimming crab (*Portunus trituberculatus*). Ecotoxicol. Environ. Saf..

[37] Dawood M.A., Amer A.A., Elbialy Z.I., Gouda A.H. (2020). Effects of including triticale on growth performance, digestive enzyme activity, and growth-related genes of Nile tilapia (*Oreochromis niloticus*). Aquaculture.

[38] Sukhovskaya I.V., Borvinskaya E.V., Smirnov L.P., Kochneva A.A. (2017). Role of glutathione in the functioning of the system of antioxidant protection in fish. Inland Water Biol..

[39] Chen Y., Dong H., Thompson D.C., Shertzer H.G., Nebert D.W., Vasiliou V. (2013). Glutathione defense mechanism in liver injury: Insights from animal models. Food Chem. Toxicol..

[40] Bordbar S., Ebrahimpour A., Hamid A.A., Manap M.Y.A., Anwar F., Saari N. (2013). The improvement of the endogenous antioxidant property of stone fish (*Actinopyga lecanora*) tissue using enzymatic proteolysis. BioMed Res. Int..

[41] Sevcikova M., Modra H., Blahova J., Dobsikova R., Plhalova L., Zitka O., Hynek D., Kizek R., Skoric M., Svobodova Z. (2016). Biochemical, haematological and oxidative stress responses of common carp (*Cyprinus carpio* L.) after sub-chronic exposure to copper. Vet. Med..

[42] Mikulikova I., Modrá H., Blahova J., Marsalek P., Groch L., Siroka Z., Kruzikova K., Jarkovsky J., Littnerová S., Svobodova Z. (2011). The effects of Click 500 SC (terbuthylazine) on common carp *Cyprinus carpio* under (sub) chronic conditions. Neuroendocrinol. Lett..

[43] Farjad E., Momeni H.R. (2018). Silymarin ameliorates oxidative stress and enhances antioxidant defense system capacity in cadmium-treated mice. Cell J..

[44] Xu H., Yang M., Qiu W., Pan C., Wu M. (2013). The impact of endocrine-disrupting chemicals on oxidative stress and innate immune response in zebrafish embryos. Environ. Toxicol. Chem..

[45] Carlström M. (2021). Nitric oxide signalling in kidney regulation and cardiometabolic health. Nat. Rev. Nephrol..

[46] Wang X., Han C., Cui Y., Li S., Jin G., Shi W., Bao Y. (2021). Florfenicol causes excessive lipid peroxidation and apoptosis induced renal injury in broilers. Ecotoxicol. Environ. Saf..

[47] Seely J.C., Francke S., Mog S.R., Frazier K.S., Hard G.C. (2019). Renal Papillary Rarefaction: An Artifact Mimicking Papillary Necrosis. Toxicol. Pathol..

[48] Razzaque M.S. (2013). Phosphate toxicity and vascular mineralization. Phosphate and Vitamin D in Chronic Kidney Disease.

[49] Reda R.M., Ibrahim R.E., Ahmed E.N.G., El-Bouhy Z.M. (2013). Effect of oxytetracycline and florfenicol as growth promoters on the health status of cultured *Oreochromis niloticus*. Egypt J. Aquat. Res..

[50] Wolf J.C., Baumgartner W.A., Blazer V.S., Camus A.C., Engelhardt J.A., Fournie J.W., Frasca S., Groman D.B., Kent M.L., Khoo L.H. (2015). Nonlesions, misdiagnoses, missed diagnoses, and other interpretive challenges in fish histopathology studies: A guide for investigators, authors, reviewers, and readers. Toxicol. Pathol..

[51] Kalra A., Yetiskul E., Wehrle C.J., Tuma F. (2018). Physiology, Liver.

[52] Inglis V., Richards R.H., Varma K.J., Sutherland I.H., Brokken E.S. (1991). Florfenicol in Atlantic salmon, *Salmo salar* L., parr: Tolerance and assessment of efficacy against furunculosis. J. Fish Dis..

[53] Straus D.L., Bowker J.D., Bowman M.P., Carty D., Mitchell A.J., Farmer B.D. (2012). Safety of aquaflor-medicated feed to sunshine bass. N. Am. J. Aquac..

